# Treatment of Pneumonia

**Published:** 1871-11

**Authors:** 


### Treatment of Pneumonia.

M. Papillaud terminates an interesting paper published in some recent numbers of the *Gazette Medicale*, on the "Treatment of Pneumonia," with the following conclusions: 1. Bleeding is hurtful in the treatment of malignant or adynamic pneumonia. 2. It is also injurious, whatever may be the form of the pneumonia, in feeble or debilitated subjects. In robust subjects it may be serviceable as an expedient for the relief of the embarrassed respiration, but opium may render the same service without producing the inconveniences consequent on the loss of blood. 3. In the treatment of pneumonia, tartar emetic is a remedy of the first rank, by reason of its antipyretic, antiphlogistic, and decongestive action; but there is no need to raise the dose beyond from 10 to 20 centi-grammes, nor to continue its employment for a longer period than from two to four days. 4. Alcoholic liquids, in doses varying from 30 to 100 grammes according to the subjects, are medical agents which, whether by aiding organic combustion, or from other as yet little known modes of action, powerfully contribute to the resolution of pneumonia. 5. A formula combining these three medicinal agents—opium, antimony, and alcohol—is that which will best meet all the ordinary indications in the treatment of pleuropneumonia.—*Medical and Surgical Journal.*

